# 

**Published:** 1872-09

**Authors:** 


### TABLE OF CONTENTS.

ORIGINAL COMMUNICATIONS.

NORMAL OVARIOTOMY—CASE. By ROBERT BATTEY, M.D.......... 321
FRACTURE OF THE SKULL, WITH LACERATION OF MEMBRANES AND INJURY TO BRAIN. By B. F. CHAMBLISS, M.D. (Read Before the Middle Georgia Medical Society.). . 339
HERNIOTOMY. By D. W. HAMMOND, M.D. (Read Before the Macon Medical Association, and Published by Order of said Body.)........ 342
MICROSCOPIC ORGANISMS AS INSTIGATORS OF DISEASE. By CH. RAUSHENBERG, M.D.... 348
VERATRUM IN PNEUMONIA. By W. H. C. BLALOCK, M.D........ 359
THE RELATION BETWEEN THE PHYSICIAN AND PHARMACEUTIST. (Reply to Z. T. Daniel, M.D.) By TH. SCHUMANN, Pharmaceutist........ 363
HINTS TO YOUNG PRACTITIONERS—NO. III. By SENEX........ 367

FOREIGN CORRESPONDENCE.

NEW OPERATION FOR THE EXTRACTION OF CATARACT. By A. W CALHOUN, M.D....... 370

EDITORIAL AND MISCELLANEOUS.

RELATION BETWEEN THE PHYSICIAN AND PHARMACEUTIST........ 376
NORMAL OVARIOTOMY........ 376
REMOVING A FOREIGN BODY FROM THE NOSE........ 377
STYPTIC COTTON........ 377

